# Voluntary running exercise protects against sepsis-induced early inflammatory and pro-coagulant responses in aged mice

**DOI:** 10.1186/s13054-017-1783-1

**Published:** 2017-08-08

**Authors:** Karel Tyml, Scott Swarbreck, Cynthia Pape, Dan Secor, James Koropatnick, Qingping Feng, Ruud A. W. Veldhuizen, Sean E. Gill

**Affiliations:** 1 Centre for Critical Illness Research, London, Ontario Canada; 2 Cancer Research Program, Lawson Health Research Institute, London, Ontario Canada; 3 Division of Respirology, University of Western Ontario, London, Ontario Canada; 4 Department of Medicine, University of Western Ontario, London, Ontario Canada; 5 Department of Physiology and Pharmacology, University of Western Ontario, London, Ontario Canada; 6 Department of Oncology, University of Western Ontario, London, Ontario Canada; 7 Department of Pathology, University of Western Ontario, London, Ontario Canada; 8 Department of Medical Biophysics, University of Western Ontario, London, Ontario Canada

**Keywords:** Aging, sepsis, Pulmonary inflammation, Capillary plugging, Voluntary running

## Abstract

**Background:**

Despite many animal studies and clinical trials, mortality in sepsis remains high. This may be due to the fact that most experimental studies of sepsis employ young animals, whereas the majority of septic patients are elderly (60 − 70 years). The objective of the present study was to examine the sepsis-induced inflammatory and pro-coagulant responses in aged mice. Since running exercise protects against a variety of diseases, we also examined the effect of voluntary running on septic responses in aged mice.

**Methods:**

Male C57BL/6 mice were housed in our institute from 2–3 to 22 months (an age mimicking that of the elderly). Mice were prevented from becoming obese by food restriction (given 70–90% of *ad libitum* consumption amount). Between 20 and 22 months, a subgroup of mice ran voluntarily on wheels, alternating 1–3 days of running with 1–2 days of rest. At 22 months, mice were intraperitoneally injected with sterile saline (control) or 3.75 g/kg fecal slurry (septic). At 7 h post injection, we examined (1) neutrophil influx in the lung and liver by measuring myeloperoxidase and/or neutrophil elastase in the tissue homogenates by spectrophotometry, (2) interleukin 6 (IL6) and KC in the lung lavage by ELISA, (3) pulmonary surfactant function by measuring percentage of large aggregates, (4) capillary plugging (pro-coagulant response) in skeletal muscle by intravital microscopy, (5) endothelial nitric oxide synthase (eNOS) protein in skeletal muscle (eNOS-derived NO is putative inhibitor of capillary plugging) by immunoblotting, and (6) systemic blood platelet counts by hemocytometry.

**Results:**

Sepsis caused high levels of pulmonary myeloperoxidase, elastase, IL6, KC, liver myeloperoxidase, and capillary plugging. Sepsis also caused low levels of surfactant function and platelet counts. Running exercise increased eNOS protein and attenuated the septic responses.

**Conclusions:**

Voluntary running protects against exacerbated sepsis-induced inflammatory and pro-coagulant responses in aged mice. Protection against pro-coagulant responses may involve eNOS upregulation. The present discovery in aged mice calls for clinical investigation into potential beneficial effects of exercise on septic outcomes in the elderly.

**Electronic supplementary material:**

The online version of this article (doi:10.1186/s13054-017-1783-1) contains supplementary material, which is available to authorized users.

## Background

Sepsis is a systemic inflammatory response to local infection, leading to multiple organ failure and 40% mortality in Intensive Care Units [1]. The prevalence of sepsis is highest in the elderly (age 60 − 70 years) where the outcome disproportionately worsens with age [2]. Despite many animal studies and more than 70 clinical trials, there is no definitive treatment for sepsis [3] and, as such, the already high socioeconomic burden of sepsis is expected to increase further with our aging population.

The development of sepsis has been studied in animal models ranging from the simple injection of lipopolysaccharide (LPS) to the more clinically relevant model of cecal ligation and perforation (CLP). Several organ systems are affected and many pathophysiological processes are involved in sepsis. For example, in the lung, sepsis often leads to acute respiratory distress syndrome and is associated with overwhelming inflammation, including neutrophil infiltration, edema formation, and alterations to the pulmonary surfactant system [4]. Inflammation in other organs systems, such as liver, heart, kidneys, and various systemic processes, such as the function of the microvascular system and the coagulation pathways, are also involved in the initiation and progression of the septic response [5].

Notably, the vast majority of animal studies of sepsis have employed relatively young animals. Because the outcome of sepsis could be aggravated by age, studies in young animals may have a limited impact on our understanding and development of therapeutic strategies to treat sepsis in the elderly. Indeed, Starr and coworkers [6] showed that LPS injected into young mice (4 months) at a dose insufficient to cause mortality at 72 h post-LPS resulted in 80% mortality in old mice (24 months). The high mortality was linked to increased sepsis-induced coagulation and to reduced antioxidant defense in aged mice [7]. Similarly, CLP-induced sepsis resulted in 100% survival of young mice at 48 h post-CLP, but only 50% survival of old mice [8]. The lower survival correlated with increased plasma levels of inflammatory cytokines in old mice [8].

Considering the complex pathophysiology of sepsis, focus on prevention rather than treatment appears to be warranted. In this context, it is well-known that running exercise offers protection across a variety of diseases, including those associated with inflammation [9, 10]. Importantly, this protection has also been reported for sepsis. For example, following a 4-week running protocol, young rats injected with LPS demonstrated reduced inflammatory responses in various organs [11]. In young mice, running exercise also protected against CLP-induced and *Streptococcus pneumoniae*-induced sepsis, and against severe polymicrobial sepsis, in the lung and other organs [12–14]. However, to our knowledge, the effect of exercise on the septic response has not been examined in aged animals. Our overall hypothesis was that exercise protects against sepsis in aged mice. This hypothesis was tested in 22-month-old mice, using voluntary running as an exercise regimen, and examining the effects on several pathophysiological processes associated with sepsis.

## Methods

### Experimental design

#### Model of aging

The age of the most frequently treated septic patients has been reported to be 65 years [15]. Based on the US Actuarial database, about 80% of the human male population reaches 65 years of age [16]. Moreover, about 80% of male wild-type C57BL/6 mice reach 22 months of age [17]. Male wild-type C57BL/6 mice were therefore aged to 22 months in our institutional animal facility under controlled conditions (i.e., standard light-dark cycle, temperature, humidity, food, and bedding). Experimental protocols, including induction of sepsis, were performed in accordance with the Canadian Council on Animal Care guidelines for the care and handling of animals. The institutional Animal Care Committee approved our protocols, except for survival studies of septic aged mice (Approval # 2011-062).

Mice presented with unrestricted regular food during aging typically become obese. Because obesity worsens the inflammatory response in septic mice [18], our aging mice were food-restricted to focus the study on the effects of aging and exercise, thus avoiding the confounding effect of obesity with age. Mice were ordered from Charles River laboratories (Sherbrooke, QC, Canada) at 2–3 months of age, in batches of 10 at approximately 1–2 month intervals. Upon arrival, mice were weighed and housed in groups of four to six per cage. Mice in the initial batch were provided with unrestricted standard food plus water, and food consumption was measured. The amount of food provided to the mice was then reduced by 10% (of the measured amount) every 2–3 weeks until reaching 70% (i.e., 2.5 g/mouse/day) of the measured amount, which was maintained for the duration of the aging. Subsequent batches of mice followed the same protocol but started at 90% of the initial measured amount [19, 20]. As found previously, under this regime, aging mice did not gain weight [19]. Mice were weighed weekly to ensure they maintained body weight during aging. If a mouse in a particular cage began losing weight (i.e., its mate(s) fed more avidly), the mouse was then housed alone to allow weight recovery and subsequent weight maintenance during aging.

#### Model of running exercise

There are numerous animal models of running exercise, including voluntary running on a wheel accessible 24 h each day [21] and treadmill running where an animal’s running is controlled [22]. In general, the intensity and duration of exercise in these models have been used at levels high enough to ensure an effect of exercise.

The present study utilized a model of running that aimed to mimic the physical activity of elderly individuals (i.e., approaching retirement) who have begun moderate but regular aerobic exercise later in life (i.e., to get health benefits from exercise when they are older). Because aging increases the potential for muscle and joint injury during exercise [23–25], these individuals may wish to include days of rest within their routine, to minimize this potential injury. Thus, between 20 and 22 months, a subgroup of mice ran voluntarily on wheels, alternating days of running with days of rest, to allow recovery from exercise. We hypothesized that the present model at submaximal duration and intensity (i.e., starting exercise later in life and including days of rest) would still yield beneficial effects against sepsis.

Specifically, at age 20 months a subset of the aging mice were put in cages with running wheels to which a miniature magnet was attached (Mouse Igloo Fast-Trac, BioServ, Flemington, NJ, USA). Each cage housed two mice and two wheels, or one mouse and one wheel, on which the mice could run voluntarily 24 h per day, and mice were monitored daily for running activities. Further, a sensor attached to the outside wall of the cage near the wheel measured the number of wheel revolutions during mouse running, permitting determination/estimation of the distance run by each mouse. The physical activity protocol consisted of (1) an initial period of 7 days when mice could run voluntarily every day, and (2) an 8-week period when 1–3 days of running were alternated with 1–2 days of recovery from running. To facilitate recovery, the wheel-igloo assembly was disengaged so that the wheel could not turn. To maintain body weight, running mice were provided with 90% (i.e., 3.2 g/mouse/day) of measured food between 20 and 22 months. Control non-running mice had no wheel in their cage; their physical activity continued to be the same as that for all mice prior to 20 months.

#### Model of sepsis

In the present study, we chose a model of sepsis involving fecal injection into the peritoneum (FIP) at the dose of 3.75 g/kg [26, 27]. Using this model of sepsis permitted comparison of our previously accumulated data in young mice at 7 h post-FIP [26, 27] with data from the present aged mice. The FIP dose in young mice resulted in 20% survival at 24 h post-FIP ([26]). Based on the reported worsening of survival when comparing young and old CLP mice [8], we predicted our aged mice would not survive past 12–14 h post-FIP at this dose. Thus, the focus of the present study in septic aged mice was on the early stage of the inflammatory response (i.e., 7 h post-FIP, as in our young mice).

Specifically, feces were collected from the cecum of a minimum of two donor mice, suspended in sterile saline at a concentration of 75 mg/mL, and stored overnight at 4 °C. The following day, mice were injected intraperitoneally with 3.75 g/kg of the feces slurry. Following feces injection, mice were fluid resuscitated by subcutaneous injection of 1 mL of sterile saline containing 4 μg/mL buprenorphine. For control sham mice, sterile saline was substituted for feces solution. In mice subjected to the running protocol, sepsis was induced 1–2 days after the last running bout.

### Intravital microscopy of skeletal muscle, collection of fluids and tissues, and biochemical analyses

Sepsis-induced inflammation leads to activation of the coagulation pathway [5]. We used the skeletal muscle as a bioassay to examine this aspect of sepsis in terms of capillary bed plugging, a well-known indicator of sepsis involving pro-coagulation responses [28]. Capillary plugging, reported in animal and human organs during sepsis, leads to inadequate oxygenation of the tissue and organ failure [28].

At 6 h post-FIP, mice were anesthetized with ketamine (80 mg/kg) and xylazine (4 mg/kg) and injected via the intrapenile vein with 0.1 mL of sterile saline. At 6.5 h post-FIP, surgery was begun to expose the right extensor digitorum longus (EDL) muscle, thereby allowing its surface to be imaged at 7 h post-FIP using intravital microscopy and epi-illumination as described previously [26, 27]. Importantly, because of the epi-illumination aspect of this microscopy, the EDL muscle was not touched by surgical tools during this process [29], resulting in little or no surgical injury to the muscle. Intravital images were used to determine capillary plugging. To this end, we measured the density of perfused capillaries and the density of stopped-flow capillaries seen within a 100-μm-deep surface layer of the muscle. Specifically, in each mouse, 4–5 fields of view of the muscle were used for determination of capillary plugging. In each field, a test line was drawn across muscle fibers, and capillaries with moving and stationary red blood cells (RBC) crossing the test line were counted. The total density was computed from the sum of capillaries with moving RBC and stationary RBC. Capillary plugging was determined as the ratio of stopped-flow to total capillaries (perfused plus stopped-flow) as previously reported [26]. The values of total densities and of capillary plugging were averaged among the 4–5 fields. During the period from 6 h post-FIP to the end of the intravital experiment at ~7.5 h post-FIP, the mice were kept anesthetized with supplemental ketamine/xylazine doses approximately every 30 min.

Shortly after the intravital study was completed, the following procedures were carried out: (1) a blood sample from a punctured carotid artery was collected into a heparinized syringe containing the anticoagulant acid citrate dextrose (25–50 μL per sample) for subsequent systemic platelet count and lactate analyses; (2) the peritoneal cavity was lavaged with 2 mL sterile saline to assess bacterial count in the peritoneal fluid; (3) bronchoalveolar lavage (BAL) was done (detailed below); and (4) the lung, liver, and left hindlimb skeletal muscle (including the EDL) were harvested and flash-frozen in liquid N_2_. Frozen tissue samples were subjected to further biochemical analyses.

To determine the systemic blood platelet count, blood was diluted 200-fold in saline, and platelets were labeled with rhodamine 6G (0.4 μg/mL, Sigma-Aldrich) or calcein-AM (8 μM, Sigma-Aldrich) and counted in a hemocytometer chamber under a microscope. Plasma lactate was determined with the iSTAT system, cartridge CG4+ (Abbott, Mississauga, ON, Canada). To determine the bacterial count, peritoneal lavage samples were serially diluted in 10-fold fashion, plated on Columbia Blood Agar containing 5% sheep blood (MP0351, Oxoid, Nepean, ON, Canada) and grown overnight at 37 °C. The bacterial colony-forming units (CFU) were counted and expressed per μL of the peritoneal lavage fluid.

The BAL procedure and processing was performed as previously reported [30]. Briefly, after securing an endotracheal tube, lungs were lavaged with three boluses of 1 mL of saline. The total lavage volume was centrifuged at 150 g for 10 min to remove cells and debris, and the supernatant was used for measurement of interleukin 6 (IL6) and KC concentrations by ELISA (BD Bioscience, San Diego, CA, USA). A separate aliquot of the supernatant was used to isolate the two sub-fractions of pulmonary surfactant, the large aggregates (LA), which are the functional components of surfactant, and the small aggregates (SA), which are inactive. The amounts of phospholipids (PL) in LA and SA were determined by phosphorus assay after an organic solvent extraction [31, 32].

#### Myeloperoxidase (MPO) assay

Tissue MPO accumulation is well-known to be associated with the degree of inflammation (i.e., neutrophil infiltration), as we have observed both in sepsis and lung injury [33, 34]. To assess lung and liver inflammation following sepsis, we examined total MPO abundance within these tissues. To this end, frozen tissues were homogenized in 20 mM potassium phosphate buffer and centrifuged at 6000 g for 20 min. After discarding the supernatant, the pellet was resuspended in 50 mM acetic acid with 0.5% hexadecyltrimethylammonium hydroxide detergent. The samples were then sonicated for 10 s and centrifuged for 5 min. The supernatants were mixed with a 1 mM tetramethylbenzidine (TMB) and 0.2 M acetic acid solution, warmed to 37 °C, and the reaction initiated with the addition of 3 mM H_2_O_2_. The samples were left to react for 5–30 min and then quenched using 1000 U/mL beef catalase and 0.2 M acetic acid. Spectrophotometric analysis (655 nm) was conducted using the Model 680 microplate reader and software version 5.2.1 (Bio-Rad), and compared to an MPO standard (catalogue # M6908; Sigma).

#### Neutrophil elastase (NE) ELISA

NE is a serine protease found primarily within the azurophilic granules of the neutrophil [35]. To confirm neutrophil influx into the lungs, we examined total NE abundance by DuoSet ELISA according to the manufacturer’s protocol (catalogue # DY4517-05; R&D Systems). Briefly, frozen lung tissue was homogenized in CellLytic M lysis buffer with proteinase inhibitor (Sigma). Wells of a 96-well plate were incubated with capture antibody overnight at room temperature and then washed three times with PBS and 0.05% Tween (PBST). Wells were blocked with PBS and 1% bovine serum albumin (BSA) for 1 h, washed with PBST, and incubated with lung sample or standard (12.5 − 800 pg/mL) overnight at 4 °C. Wells were again washed with PBST and then incubated with detection antibody. After 2 h, wells were washed with PBST and then incubated with streptavidin conjugated to horseradish peroxidase (HRP) for 20 min. Wells were once again washed with PBST, and TMB substrate was then added to each well and incubated for 20 min. The reaction was stopped using 1 M H_2_SO_4_ and plates read at 450 nm using the Model 680 microplate reader and software version 5.2.1 (Bio-Rad).

#### Immunoblotting of endothelial nitric oxide synthase (eNOS)

A total of 50 μg protein from the left skeletal muscle was loaded and separated by 10% SDS-PAGE, followed by electrotransfer to a nitrocellulose membrane. The blots were probed with specific primary antibodies against mouse eNOS (BD Transduction Laboratories) and glyceraldehyde 3-phosphate dehydrogenase (GAPDH; Sigma). Each band was visualized by enhanced chemiluminescence detection and exposure to X-ray film. Optical density for individual bands was examined using the FluorChem 8000 (Alpha Innotech, San Leandro, CA, USA). The densitometry ratios of eNOS to GAPDH were then computed.

#### Heart function

In some aged mice, the heart rate (HR) and cardiac output (CO) were examined at 6 h after FIP or sham injection. Mice were lightly anesthetized with 1–2% isoflurane, and HR and CO measured using the Vevo 2100 ultrasound imaging system (Visual Sonics, Canada) as previously reported [36–38].

### Mouse attrition/usage

From 69 mice delivered to our institution at 2–3 months of age, 63 mice reached 22 months (i.e., 91% survival). However, 20 of these mice were excluded from the experimental analyses because of an erroneous feces dose injected (7 mice), death during intravital microscopy caused by ketamine/xylazine overdose (6 mice), tumors found in the liver (3 mice), and an unidentified illness/lethargy in the mice (4 mice).

### Statistical analysis

Data are presented as mean ± SEM. Statistical analysis was carried out using the Student *t* test or two-way analysis of variance (ANOVA) with the Bonferroni multiple comparison test. Results were considered significant at *P* < 0.05.

## Results

### Body weight, running distance, and effects of sepsis and exercise on lactate and bacterial count

Mouse body weight at 22 months (26.6 ± 0.4 g, n = 43) was comparable to that at 2–3 months (25.3 ± 0.3 g, n = 69). Further, there was no difference in body weight between non-running and running control mice at 22 months. All mice housed with engaged wheels were observed to run on the wheels at all times. On average, mice ran 43.4 ± 0.7 days at 7.5 ± 0.6 km/day (n = 16) from 20 to 22 months. Sepsis increased lactate and peritoneal fluid bacterial count in comparison to control mice; there was no difference in lactate and bacterial count between non-running and running mice among either the control or septic aged mice (Table 1).

**Table 1:** Historical comparison between outcomes after 7 h of sepsis in young (1–4 months) and old (22 months) mice, based on our published data in young mice and the present study in aged mice

Septic outcome	Young		Old	
	Control	Septic	Control	Septic
Feces dose (g/kg)	-	3.75 [26]	-	3.75
Lactate (mM)	0.7 ± 0.3 n = 6 [26]	2.1 ± 0.4 n = 6 [26]	0.49 ± 0.09 n = 8	1.87 ± 0.26 n = 6
Peritoneal fluid bacterial count (CFU)	0.30 ± 0.26 n = 13 [33]	44,520 ± 19,060 n = 15 [33]	1.6 ± 1.6 n = 9	32,066 ± 14,791 n = 7
Lung MPO (U/mL/mg)	0.16 ± 0.03 n = 10 [33]	0.41 ± 0.07 n = 9 [33]	1.66 ± 0.43 n = 6	4.28 ± 0.33 n = 6
Liver MPO (U/mL/mg)	0.023 ± 0.005 n = 6 [33]	0.071 ± 0.009 n = 6 [33]	0.35 ± 0.04 n = 6	0.60 ± 0.04 n = 6
Capillary plugging (%)	7.7 ± 0.6 n = 18 [27]	41.0 ± 1.5 n = 16 [27]	9.1 ± 1.0 n = 9	80.0 ± 8.8 n = 7

**Fig. 1:**
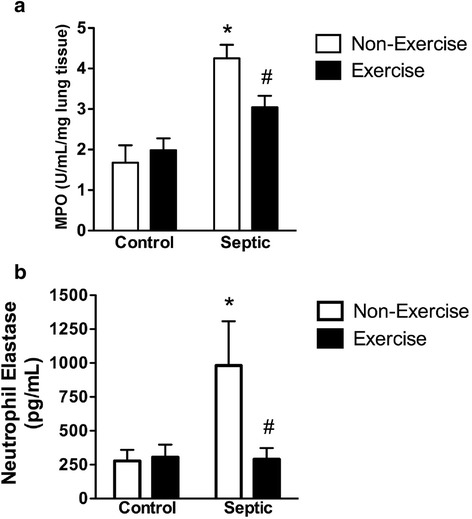
Effect of sepsis and running exercise on myeloperoxidase (MPO) and neutrophil elastase (NE), measures of inflammatory neutrophil infiltration, in lung homogenates of aged mice (22 months). Sepsis induced by fecal injection into the peritoneum (7 h post-injection) increased lung MPO (**a**) (n = 6, 5, 6, and 6 for *bars left* to *right*, respectively) and NE (**b**) (n = 4 per *bar*) in non-exercised mice. Running exercise protected against these increases. *Effect of sepsis versus control, ^#^effect of exercise versus non-exercise; *P* < 0.05

Data are presented as mean ± SE. Data for young mice are from our previous publications as indicated (Tyml et al., [26]; Swarbreck et al., [33]; Secor et al., [27]). Data for old mice lung and liver myeloperoxidase (MPO) and capillary plugging are from Figs. 1a, 4 and 5b, respectively.

### Septic pulmonary and non-pulmonary responses

**Fig. 2:**
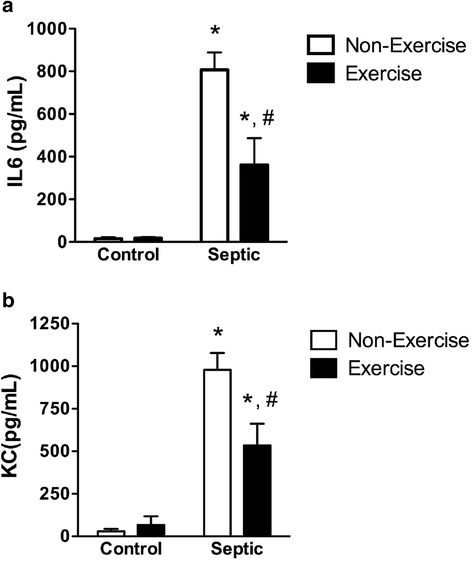
Effect of sepsis and running exercise on concentrations of interleukin 6 (IL6) (**a**) and KC (**b**) in lung lavage of aged mice: n = 6, 5, 6, and 6 for *bars*, *left* to *right*, respectively (**a** and **b**). Sepsis (7 h post fecal injection into the peritoneum) increased lung IL6 and KC in both exercised and non-exercised mice. Running exercise reduced these increases. *Effect of sepsis versus control, ^#^effect of running exercise versus non-exercise; *P* < 0.05

In non-running mice, sepsis elevated pulmonary MPO compared to control. In running mice, this elevated level was reduced (Fig. 1a). Similar responses were observed in pulmonary NE, a complementary measure of neutrophil infiltration (Fig. 1b). Sepsis also increased IL6 and KC levels in non-running mice, and these increased levels were reduced in running mice (Fig. 2a, b).

**Fig. 3:**
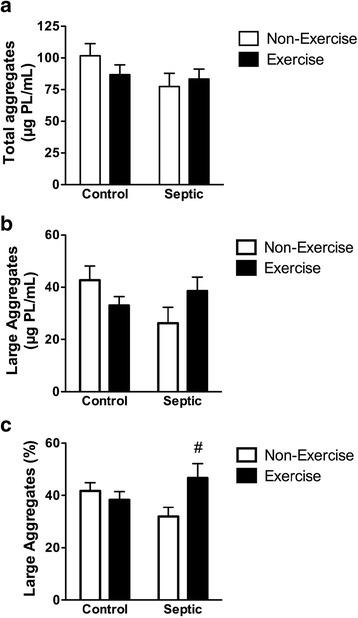
Effect of sepsis and running exercise on surfactant pool size in lung lavage from aged mice. **a** The total was the sum of functional large aggregates and the inactive small aggregates of phospholipids (PL) measured in the bronchoalveolar lavage fluid. The large aggregate (**b**) and small aggregate sub-fractions were measured to assess the large aggregate percentage (**c**), a measure of the relative amount of functional lung surfactant. ^#^Effect of running exercise versus non-exercise; *P* < 0.05: n = 6, 5, 6, and 6 for *bars*, *left* to *right*, respectively (**a**, **b**, **c**)

With regards to the surfactant system, there were no differences in total surfactant between any groups (Fig. 3a); however, the amount of large aggregates appeared to be reduced in septic non-running mice and this decrease was not observed in septic running mice (Fig. 3b). The percentage of large aggregates with respect to total surfactant in septic non-running mice also appeared to be decreased compared to control mice but this difference was not significant (Fig. 3c). However, the percentage of large aggregates was significantly increased in septic running mice versus septic non-running mice (Fig. 3c).

**Fig. 4:**
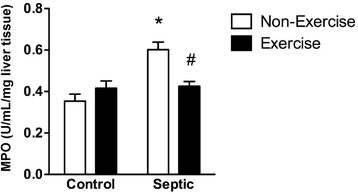
Effect of sepsis and running exercise on myeloperoxidase (*MPO*) in liver homogenates of aged mice. Sepsis (7 h post fecal injection into the peritoneum) increased liver MPO in non-exercised mice. Running exercise protected against this increase. *Effect of sepsis versus control, ^#^effect of running exercise versus non-exercise; *P* < 0.05, n = 6 per *bar*

Sepsis also elevated liver MPO compared to control mice (Fig. 4). Further, similar to our observations in lung tissue, prior running reduced this elevation in liver MPO accumulation (Fig. 4). In addition, sepsis lowered HR and CO compared to control mice (Additional file 1: Figure S1), a finding consistent with the literature [39, 40]. However, no differences were observed between running and non-running controls or septic mice (Additional file 1: Figure S1).

### Septic capillary plugging in skeletal muscle

**Fig. 5:**
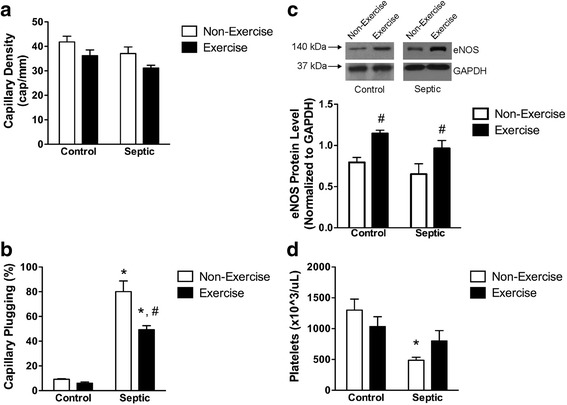
Effect of sepsis and running exercise on capillary bed plugging in the extensor digitorum longus muscle, endothelial nitric oxide synthase (*eNOS*) protein, and systemic arterial blood platelet count, in aged mice. **a** Capillary density in the extensor digitorum longus muscle was computed from the sum of capillaries with moving red blood cells (RBC) and stationary RBC. There was no effect of sepsis or exercise on total capillary density: n = 9, 6, 7, and 6 for *bars*, *left* to *right*, respectively. **b** Capillary plugging, computed as the percentage of capillaries with stationary RBC from the total of capillaries (perfused plus stationary RBC capillaries). Sepsis (7 h post fecal injection into the peritoneum (FIP)) increased plugging in both exercised and non-exercised mice. Running exercise reduced this increase. *Effect of sepsis versus control, ^#^effect of running exercise versus non-exercise; *P* < 0.05: n = 9, 6, 7, and 6 for *bars*, *left* to *right*, respectively. **c** Sepsis (7 h post-FIP) did not significantly alter eNOS levels assessed by immunoblot. Running exercise, however, significantly increased eNOS in skeletal muscle of both controls and septic mice: ^#^effect of running exercise versus non-exercise; *P* < 0.05; n = 4 per *bar*. **d** Sepsis (7 h post-FIP) reduced platelet count in non-exercised mice, but not in exercised mice: *effect of sepsis versus control; *P* < 0.05; n = 9, 6, 7, and 6 for *bars*, *left* to *right*, respectively

There were no differences in the total capillary density between any groups (Fig. 5a). In non-running mice, sepsis increased capillary plugging (Fig. 5b; Additional files 2, 3, 4 and 5: Videos V1-V4). In running mice, however, this increased plugging was reduced (Fig. 5b; Additional files 2, 3, 4 and 5: Videos V1-V4).

Exogenous NO reduces sepsis-induced capillary plugging in skeletal muscle [27]. Further, eNOS-derived NO reduces platelet-endothelial adhesion [41] and this adhesion is a major mechanism for capillary plugging in sepsis [27]. Immunoblots from muscle homogenates revealed upregulated eNOS in both control and septic running mice versus non-running mice (Fig. 5c).

Platelet counts (Fig. 5d) complemented capillary plugging data. Plugging involves platelet trapping in capillaries [27] leading to a subsequent reduction in the number of platelets available for detection in systemic blood [42]. Sepsis led to a significantly decreased platelet count versus controls in non-running mice (Fig. 5d). Although platelet counts were not different between septic non-running and running mice, unlike in non-running mice, platelet counts were not reduced in septic running versus control running mice (Fig. 5d).

## Discussion

### Effect of sepsis and running exercise on inflammatory/pro-coagulant responses

In mice at 22 months, inflammatory/coagulation responses in the lung, liver, and skeletal muscle capillary bed were markedly increased after 7 h of sepsis when compared to control. We show for the first time that running exercise started at 20 months, and including 1–2 days of rest between runs, protects against these early septic responses in aged mice. Further, the septic inflammatory responses were observed at a location remote to the original site of infection, and therefore they can be considered a pathophysiological process, suggesting the effect of running exercise was indeed protective [43]. Our findings are consistent with the reports that inadequate exercise in humans is a risk factor for sepsis mortality [44] and that running exercise protects against sepsis in young mice [12–14].

### Mechanism of protection by exercise

Most mechanistic studies of the beneficial effects of exercise were done in the skeletal muscle. Mechanisms of protection against the loss of function/structure of aging muscle have been reviewed recently and include improved mitochondrial biogenesis and autophagy, PGC1α activation, reduced oxidative stress, and reduced background inflammation [45, 46]. Cardiovascular protection by exercise has also been reviewed [47, 48]. Interestingly, our data on HR and CO from the hearts of aged septic mice (Additional file 1: Figure S1) suggest lack of protection by exercise. However, as sepsis at 7 h does not increase MPO in the heart [34], we speculate that protection by exercise may not have manifested in the heart in contrast to protection in immunogenic organs where neutrophils become abundant (i.e., lung and liver; Figs. 1 and 4).

One mediator of the beneficial effect of exercise in skeletal muscle is eNOS [49]. Exercise-induced upregulation of eNOS, caused mainly by increases in blood flow and shear stress [50, 51], was seen in the skeletal muscle microvasculature [52], the lung, and other tissues [50, 53, 54]. eNOS-derived NO is anti-coagulatory (i.e., reduces platelet-endothelial adhesion [41]), and may participate in the reduction of capillary plugging in septic skeletal muscle [27]. Our data support this role of eNOS and suggest that the observed protection against capillary plugging in septic muscle (Fig. 5b) likely involves eNOS. While upregulated eNOS has been associated with increased eNOS enzymatic activation and NO release [50], the mechanism of reduced capillary plugging involving eNOS may be complex and warrants further investigation.

Little is known about the exercise-induced protection against inflammation in organs remote from the contracting skeletal muscle (e.g., lung or liver). Potential protective mechanisms include myokines released from contracting muscle, and an exercise-induced reduction in immunosenescence [55–57]. It has been proposed that following endurance exercise, damage-associated molecule patterns (DAMPs) leaking from the skeletal muscle to the blood stream could stimulate neutrophils to counter-regulate the acute innate immune response to avoid excessive inflammation [58]. Our data, showing that exercise attenuated sepsis-induced pulmonary and liver inflammation, are consistent with this proposal. Future studies, however, are warranted to mechanistically explain the remote protection by exercise against sepsis.

### Effect of age on inflammatory/pro-coagulant responses

The present model of sepsis permitted historical comparison between the present septic responses in aged mice and our published observations in young mice. An identical septic insult in young mice comparably increased peritoneal bacterial count and plasma lactate as in aged mice (Table 1). Table 1 also shows that lung and liver MPO levels in aged mice were much higher than those in young mice. Thus, aging itself may elevate neutrophil infiltration in both the non-septic and septic lung and liver. This age-induced worsening of the inflammatory response agrees with reported age-induced increases in the pulmonary inflammatory responses in LPS-injected mice [7] and mice that have undergone cecal ligation and perforation (CLP) [8].

There was comparable capillary plugging of 8–9% in the muscle of control non-septic aged mice and control young mice (Table 1). Septic insult (3.75 g/kg) resulted in 41% plugging in young mice (Table 1). The 80% plugging observed in septic non-running mice (Table 1) suggests that aging worsened this plugging, agreeing with reported augmented septic coagulation response with aging [59].

### Limitations of the study

The number of aged mice available for present study was limited, mainly because of the expense associated with housing mice to 22 months under controlled conditions. Thus, we did not have enough mice to examine the effect of duration, onset of exercise, sex, or other relevant factors on the outcome of sepsis. Further, we could not completely characterize our aged mice (e.g., body mass composition, glucose tolerance test, maximal oxygen uptake), or their response to sepsis (e.g., different time points of sepsis, biochemical endpoints, or tissue injury). As we did not examine different time points following sepsis, it is possible that exercise did not completely block the inflammatory/procoagulant responses during sepsis, but only delayed the onset. Thus, future work examining these responses, and tissue injury and mouse survival, at different time points, would be important to further demonstrate protection by exercise against sepsis.

Despite controlled FIP septic insult, the present model produced variable peritoneal fluid bacterial counts at 7 h of sepsis. This variability led to mouse-to-mouse variability in responses to sepsis, which, in turn, could account for the variability of data in the present study and lack of statistical significance in some of the observed trends.

## Conclusions

Voluntary running protected against exacerbated sepsis-induced inflammatory and pro-coagulant responses in mice aged to 22 months, even when including 1–2 days of rest between runs. Protection against pro-coagulant responses may involve eNOS upregulation.

The present study mimicked sepsis in the elderly, some of whom have begun moderate but regular aerobic exercise later in life. We conclude that the outcome of sepsis in the elderly would be far more severe than in the young, and suggest that regular exercise, even if begun later in life, will temper the increased severity of the septic response.

## Additional files

Additional file 1: Figure S1. Effect of sepsis and voluntary running exercise on heart rate and cardiac output of aged mice. Sepsis (6 h post-FIP) significantly decreased both heart rate (*left panel*) and cardiac output (*right panel*); however, running exercise had no observable effect. *Effect of sepsis versus control, *P* < 0.05. For each panel, n = 3, 3, 8 and 5 for *bars*, *left* to *right*, respectively. (TIF 172 kb)

Additional file 2: Video V1. An example of an intravital video recording from a microscopic field of view of hind limb skeletal muscle in a control mouse. The right extensor digitorum muscle (EDL) was surgically exposed, covered by a glass coverslip, epi-illuminated by light passed through a green filter, and viewed under a low magnification to visualize capillaries. The video camera mounted on top of the microscope was rotated until the majority of capillaries appeared in horizontal direction, along the muscle fibers. The analysis of capillary stoppage (i.e., counting the number of capillaries with moving and stationary red blood cells crossing a vertical center line drawn on the video monitor) was carried out on-line, rather than off-line from video recordings, because the recording process deteriorated the quality of the optical image of capillaries. In general, the vertical line was drawn in the center part of the field as the regions near the top and bottom edges of the field could not always be brought into focus. The counting was done over a 1-min period. If necessary, the presence of a particular capillary in the center part of the field was ascertained by gentle focusing up and down during the counting. The example shows a very low capillary stoppage in the control mouse (i.e., the number of capillaries with stationary red cells was much smaller than those with moving red cells). The width of the field of view is 835 μm. (MP4 7607 kb)

Additional file 3: Video V2. An example of a video recording from a field of view at the EDL muscle surface in a control + exercise mouse. The muscle was prepared, viewed and analyzed for capillary stoppage as described in Additional file 2: Video V1. The example shows a very low capillary stoppage in the control + exercise mouse. The field width is 835 μm. (MP4 5858 kb)

Additional file 4: Video V3. An example of a video recording from a field of view at the EDL muscle surface in a septic mouse. The muscle was prepared, viewed and analyzed for capillary stoppage as described in Additional file 2: Video V1. The example shows a high capillary stoppage in the septic mouse; the number of capillaries with stationary red cells was much larger than those with moving red cells (in the right middle part of the field, a capillary with moving red cells can be seen; the capillary branches from the slowly perfused arteriole, center). A micro-hemorrhage can be observed in the upper part of the field (a rare observation in septic mice). The field width is 835 μm. (MP4 4607 kb)

Additional file 5: Video V4. An example of a video recording from a field of view at the EDL muscle surface in a septic + exercise mouse. The muscle was prepared, viewed and analyzed for capillary stoppage as described in Additional file 2: Video V1. The example shows a moderate stoppage in the septic + exercise mouse. Capillaries with moving red cells can be seen in the middle and upper part of the field. The field width is 835 μm. (MP4 5749 kb)

## Abbreviations

BAL: Bronchoalveolar lavage
BSA: Bovine serum albumin
CFU: Colony-forming units
CLP: Cecal ligation and perforation
CO: Cardiac output
DAMPs: Damage-associated molecule patterns
EDL: Extensor digitorum longus
ELISA: Enzyme-linked immunosorbent assay
eNOS: Endothelial nitric oxide synthase
FIP: Fecal injection into the peritoneum
GAPDH: glyceraldehyde 3-phosphate dehydrogenase
HR: Heart rate
IL6: Interleukin 6
LA: Large aggregates
LPS: Lipopolysaccharide
MPO: Myeloperoxidase
NE: Neutrophil elastase
NO: Nitric oxide
PBS: Phosphate-buffered saline
PBST: Phosphate-buffered saline and 0.05% Tween
PL: Phospholipids
RBC: Red blood cells
SA: Small aggregates
TMB: Tetramethylbenzidine
