# Supplementary figures and images for: Voluntary running exercise protects against sepsis-induced early inflammatory and pro-coagulant responses in aged mice
Source: Crit Care. 2017 Aug 8;21:210. doi: 10.1186/s13054-017-1783-1 (PMC5549433; doi:10.1186/s13054-017-1783-1)

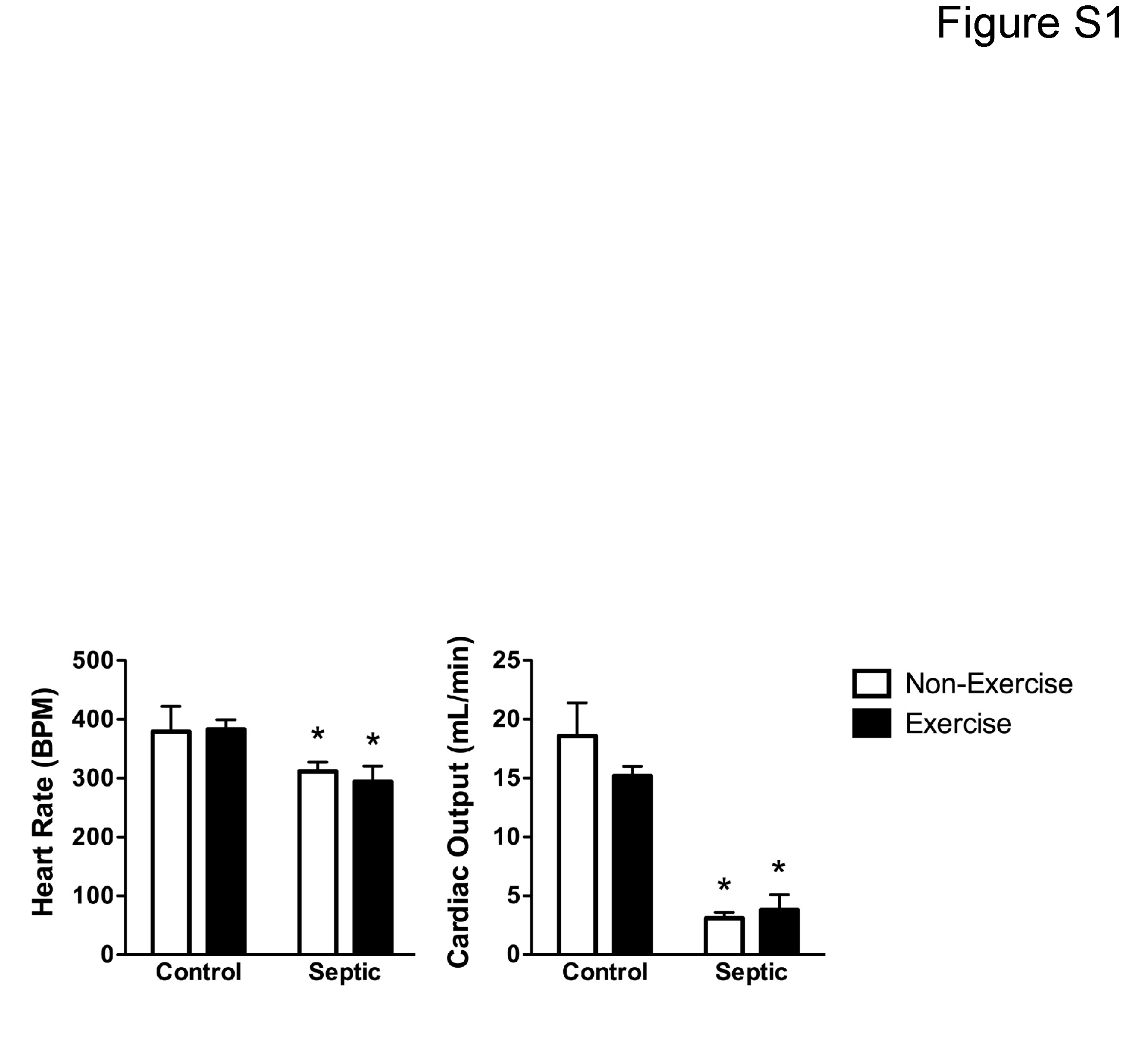

Supplement: Supplementary file 1 — Effect of sepsis and voluntary running exercise on heart rate and cardiac output of aged mice. Sepsis (6 h post-FIP) significantly decreased both heart rate (left panel) and cardiac output (right panel); however, running exercise had no observable effect. *Effect of sepsis versus control, P < 0.05. For each panel, n = 3, 3, 8 and 5 for bars, left to right, respectively. (TIF 172 kb) [file 13054_2017_1783_MOESM1_ESM.tif]
